# Overlapping mouse subcongenic strains successfully separate two linked body fat QTL on distal MMU 2

**DOI:** 10.1186/s12864-014-1191-8

**Published:** 2015-01-23

**Authors:** Rodrigo Gularte-Mérida, Charles R Farber, Ricardo A Verdugo, Alma Islas–Trejo, Thomas R Famula, Craig H Warden, Juan F Medrano

**Affiliations:** Department of Animal Science, University of California, Davis, CA 95616-8521 USA; Center for Public Health Genomics, University of Virginia, Charlottesville, VA 22908 USA; Rowe Genetics Program and Department of Pediatrics, University of California, Davis, CA 95616-8521 USA; Current Address: Unit of Animal Genomics, GIGA – Research, Avenue de l’Hôpital 1, 4031 Sart-Tilman, Belgique; Current Address: Programa de Genética Humana ICBM, Facultad de Medicina, Universidad de Chile, Independencia 1027, Santiago, Chile

## Abstract

**Background:**

Mouse chromosome 2 is linked to growth and body fat phenotypes in many mouse crosses. With the goal to identify the underlying genes regulating growth and body fat on mouse chromosome 2, we developed five overlapping subcongenic strains that contained CAST/EiJ donor regions in a C57BL/6J^*hg/hg*^ background (*hg* is a spontaneous deletion of 500 Kb on mouse chromosome 10). To fine map QTL on distal mouse chromosome 2 a total of 1,712 F2 mice from the five subcongenic strains, plus 278 F2 mice from the HG2D founder congenic strain were phenotyped and analyzed. Interval mapping (IM) and composite IM (CIM) were performed on body weight and body fat traits on a combination of SNP and microsatellite markers, which generated a high-density genotyping panel.

**Results:**

Phenotypic analysis and interval mapping of total fat mass identified two QTL on distal mouse chromosome 2. One QTL between 150 and 161 Mb, *Fatq2a*, and the second between 173.3 and 175.6 Mb, *Fatq2b*. The two QTL reside in different congenic strains with significant total fat differences between homozygous *cast/cast* and *b6/b6* littermates. Both of these QTL were previously identified only as a single QTL affecting body fat, *Fatq2*. Furthermore, through a novel approach referred here as replicated CIM, *Fatq2b* was mapped to the *Gnas* imprinted locus.

**Conclusions:**

The integration of subcongenic strains, high-density genotyping, and CIM succesfully partitioned two previously linked QTL 20 Mb apart, and the strongest QTL, *Fatq2b,* was fine mapped to a ~2.3 Mb region interval encompassing the *Gnas* imprinted locus.

**Electronic supplementary material:**

The online version of this article (doi:10.1186/s12864-014-1191-8) contains supplementary material, which is available to authorized users.

## Background

Mouse mutant models have been extensively used to identify candidate genes regulating complex traits, such as growth and body fat [1-4], providing insight into the metabolic pathways regulating these traits. In the present study we used the *high growth* (*hg*) mouse mutant model in combination with chromosome 2 (MMU2) congenic and subcongenic strains to map genes that affect body composition, and genes that may interact with the Growth Hormone/IGF signaling pathways [5-7]. Briefly, *high growth* mice are 30-50% larger than wild type littermates with body composition and organ weights proportional to their body weight. These differences in growth rate are due to a spontaneous deletion of three genes (*Socs2, Raidd/Cradd* and *Plexin C1*) on mouse chromosome 10. The key gene regulating growth in this mouse model is *Socs2,* which is part of the Jak/Stat signaling pathway and mediates the actions of Growth Hormone/IGF pathways.

Major QTL affecting body weight (*Wg2)*, carcass ash (*Cara1*) and carcass protein (*Carp1*) were previously identified in our laboratory on mouse chromosome 2 between 115 Mb and 150 Mb using a C57BL/6J^*hg/hg*^ (HG) × CAST/EiJ (CAST) F2 intercross [2] —*“hg”* refers to the *hg locus, i.e.* the deletion itself; whereas HG refers to the *high growth* mouse on a C57BL/6J background, *i.e.* C57BL/6J^*hg/hg*^. Mouse chromosome 2 has a high density of genes, many affecting relevant metabolic processes such as appetite (*Agouti, Mc3r, Scg5*), energy expenditure and storage (*Pcsk2, Atp5e, Hnf4α, Pck1*), secondary signaling cascades, and transport proteins (*Gnas, Rab22a, Vapb*). The high density of plausible functional candidate genes makes it difficult to ascertain if the QTL is the product of a single gene with one large effect, or the combined effect of several genes in close proximity each with a small effect. Thus, to further study MMU2, congenic strains were developed by introgressing CAST/EiJ segments into the backgrounds of HG and C57BL/6J (B6) to fine map QTL identified by Corva et al. and Farber et al. [2,8]. Among the MMU2 congenics developed in our laboratory, the HG.CAST–(*D2Mit329-D2Mit457*) (HG2D) is of particular importance as it contains *Fatq1,* and *Fatq2*, *Wg2, Wg5* and *Wg6* QTL within its donor region [2,8]. This strain was used to develop a panel of subcongenic strains on an HG background whose donor regions target the peaks of these previously identified QTL.

Here we report a significant improvement in the mapping resolution of the *Fatq2* QTL using the combined data from five overlapping CAST subcongenic strains and the data from a HG2D F2 intercross [9] which provides strong evidence that *Fatq2* is the result of at least two independent QTL more or less 20 Mb apart in mice homozygous for *hg*. Furthermore, we report an integrative approach used to fine map one of these QTL, *Fatq2b,* to a critical interval of 2.3 Mb containing the “*Gnas* imprinted locus” on distal MMU2.

## Results

**Figure 1:**
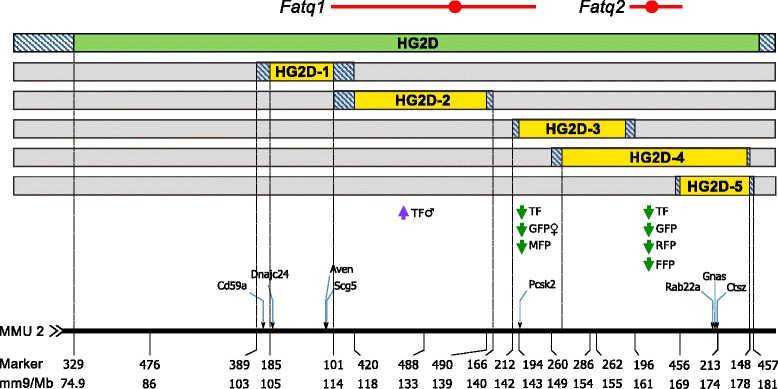
**HG2D Mouse Chromosome 2 congenic regions and phenotypic summary.** The panel of HG2D-subcongenic strains was developed by selecting recombinant mice from an HG2D F2 intercross (HG2D donor region shown in green). The top horizontal lines in red represent body fat QTL identified previously in the HG2D F2 (*Fatq1*, *Fatq2*) (QTL peak is indicated by a circle) [9]. Solid boxed areas represent regions with known alleles (green or yellow = CAST/EiJ, and gray = C57BL/6J). Textured areas correspond to recombinant ends with unknown genotype. Below the five HG2D subcongenics the arrows indicate the direction of the *additive* genetic effects of the CAST alleles at distinct genomic intervals on each fat pad trait. These analyses were conducted in each subcongenic separately. See Additional file 1 for more details. Markers, locations on mm9 and putative candidate genes are shown below on the black horizontal line, corresponding to MMU 2, blue arrows indicate the approximate location of the genes.

A graphical overview of the congenic phenotypic effects is presented in Figure 1, showing the location of the CAST allele donor regions on both the founder congenic (HG2D) and five subcongenic strains. The figure also summarizes the significant phenotypes from each subcongenic (LS Means and CAST allelic effects are found in the Table 1), and the location of body fat QTL previously mapped by Farber and Medrano in the HG2D founder strain [9]. Unless stated in the legend and on the *x-*axis, all figures display results between 74.9 to 181 Mb on mouse chromosome 2, which represents the CAST donor region in the HG2D founder congenic.

**Table 1:** **LS means for total fat, and fat pad weights for each of the 5 subcongenics**

***Strain***	***Sex*** ^***1***^	***Genotype*** ^***2***^	***N***	***RFP (g)***	***MFP (g)***	***GFP (g)***	***FFP (g)***	***TF (g)***
**HG2D-1**	**F**	**B**	53	0.102 ± 0.006	0.292 ± 0.008	0.562 ± 0.022	0.463 ± 0.014	1.419 ± 0.045
		**H**	102	0.095 ± 0.006	0.296 ± 0.008	0.544 ± 0.021	0.443 ± 0.013	1.378 ± 0.043
		**C**	40	0.101 ± 0.008	0.295 ± 0.010	0.542 ± 0.027	0.451 ± 0.017	1.389 ± 0.056
	**M**	**B**	30	0.113 ± 0.008	0.266 ± 0.011	0.453 ± 0.029	0.383 ± 0.018	1.216 ± 0.060
		**H**	88	0.122 ± 0.006	0.254 ± 0.009	0.462 ± 0.023	0.381 ± 0.014	1.219 ± 0.046
		**C**	30	0.124 ± 0.008	0.268 ± 0.010	0.468 ± 0.027	0.387 ± 0.017	1.247 ± 0.056
		***a***		*0.000 ± 0.003*	*0.002 ± 0.004*	*−0.003 ± 0.010*	*−0.003 ± 0.006*	*−0.003 ± 0.021*
		***d***		*−0.003 ± 0.004*	*−0.004 ± 0.005*	*−0.005 ± 0.014*	*−0.010 ± 0.009*	*−0.021 ± 0.028*
**HG2D-2**	**F**	**B**	33	0.092 ± 0.007	0.306 ± 0.012	0.467 ± 0.024	0.401 ± 0.015	1.267 ± 0.052
		**H**	71	0.095 ± 0.006	0.314 ± 0.011	0.466 ± 0.022	0.413 ± 0.014	1.288 ± 0.046
		**C**	20	0.095 ± 0.008	0.311 ± 0.013	0.461 ± 0.027	0.399 ± 0.017	1.266 ± 0.057
	**M**	**B**	18	0.094 ± 0.010	0.228 ± 0.017	0.370 ± 0.034	0.332 ± 0.021	1.023 ± 0.072
		**H**	45	0.104 ± 0.009	0.242 ± 0.014	0.424 ± 0.029	0.349 ± 0.018	1.118 ± 0.062
		**C**	21	0.112 ± 0.009	0.262 ± 0.014	0.437 ± 0.030	0.358 ± 0.018	1.168 ± 0.063
		***a***		*0.005 ± 0.003*	*0.009 ± 0.005*	*0.012 ± 0.010*	*0.005 ± 0.006*	*0.000 ± 0.028 ♀*
								*0.072 ± 0.032 ♂*
		***d***		*0.001 ± 0.004*	*0.001 ± 0.006*	*0.007 ± 0.013*	*0.008 ± 0.008*	*0.022 ± 0.036 ♀*
								*0.022 ± 0.044 ♂*
**HG2D-3**	**F**	**B**	29	0.096 ± 0.005	0.301 ± 0.009	0.429 ± 0.014	0.471 ± 0.012	1.395 ± 0.037
		**H**	108	0.085 ± 0.004	0.292 ± 0.008	0.395 ± 0.008	0.461 ± 0.011	1.333 ± 0.034
		**C**	40	0.083 ± 0.005	0.282 ± 0.010	0.375 ± 0.013	0.442 ± 0.013	1.281 ± 0.042
	**M**	**B**	50	0.071 ± 0.004	0.208 ± 0.008	0.402 ± 0.013	0.328 ± 0.011	0.962 ± 0.035
		**H**	98	0.067 ± 0.004	0.197 ± 0.007	0.391 ± 0.010	0.318 ± 0.009	0.926 ± 0.029
		**C**	27	0.067 ± 0.004	0.193 ± 0.008	0.416 ± 0.019	0.315 ± 0.011	0.945 ± 0.035
		***a***		*−0.004 ± 0.002*	***−0.010 ± 0.003***	***−0.032 ± 0.010 ♀***	*−0.010 ± 0.005*	***−0.039 ± 0.015***
						*0.005 ± 0.012 ♂*		
		***d***		*−0.002 ± 0.002*	*0.000 ± 0.004*	*−0.004 ± 0.013 ♀*	*0.000 ± 0.006*	*−0.008 ± 0.019*
						*−0.016 ± 0.015 ♂*		
**HG2D-4**	**F**	**B**	18	0.068 ± 0.005	0.270 ± 0.016	0.484 ± 0.026	0.386 ± 0.013	1.090 ± 0.041
		**H**	59	0.063 ± 0.003	0.262 ± 0.013	0.438 ± 0.022	0.352 ± 0.007	0.995 ± 0.023
		**C**	26	0.050 ± 0.005	0.253 ± 0.017	0.410 ± 0.028	0.322 ± 0.011	0.887 ± 0.035
	**M**	**B**	34	0.101 ± 0.004	0.243 ± 0.011	0.444 ± 0.018	0.379 ± 0.009	1.188 ± 0.031
		**H**	46	0.076 ± 0.004	0.221 ± 0.012	0.380 ± 0.020	0.345 ± 0.008	1.056 ± 0.027
		**C**	28	0.070 ± 0.004	0.217 ± 0.013	0.366 ± 0.022	0.324 ± 0.010	1.008 ± 0.033
		***a***		***−0.012 ± 0.002***	*−0.011 ± 0.005*	***−0.039 ± 0.008***	***−0.028 ± 0.005***	***−0.090 ± 0.017***
		***d***		*−0.003 ± 0.003*	*−0.004 ± 0.007*	*−0.018 ± 0.011*	*−0.006 ± 0.008*	*−0.029 ± 0.024*
**HG2D-5**	**F**	**B**	51	0.101 ± 0.005	0.314 ± 0.009	0.510 ± 0.018	0.457 ± 0.012	1.381 ± 0.039
		**H**	90	0.097 ± 0.004	0.307 ± 0.008	0.493 ± 0.016	0.439 ± 0.010	1.336 ± 0.033
		**C**	45	0.092 ± 0.005	0.294 ± 0.009	0.464 ± 0.018	0.416 ± 0.012	1.265 ± 0.039
	**M**	**B**	34	0.083 ± 0.006	0.240 ± 0.010	0.386 ± 0.020	0.340 ± 0.013	1.049 ± 0.043
		**H**	77	0.080 ± 0.005	0.226 ± 0.009	0.377 ± 0.019	0.335 ± 0.012	1.018 ± 0.039
		**C**	*42*	0.071 ± 0.006	0.229 ± 0.010	0.345 ± 0.020	0.314 ± 0.013	0.959 ± 0.043
		***a***		***−0.005 ± 0.002***	*−0.008 ± 0.003*	***−0.022 ± 0.007***	***−0.018 ± 0.004***	***−0.052 ± 0.014***
		***d***		*0.002 ± 0.003*	*−0.003 ± 0.005*	*0.008 ± 0.009*	*0.005 ± 0.006*	*0.013 ± 0.020*

Significant (p ≤ 0.01) additive (*a*) and dominance (*d*) effects are shown in **bold**. 1: F = Female M = Male. 2: Genotypes for the congenic donor region as B = *b6/b6 ,* H = *b6/cast* , C = *cast/cast*. If an sex × *additive* interaction was significant we provide both male and female *a* and *d* where, ♀: Female specific parameter. ♂: Male specific parameter.

### QTL partitioning and fine mapping analyses

#### QTL partitioning of body fat mass to individual subcongenic regions

**Figure 2:**
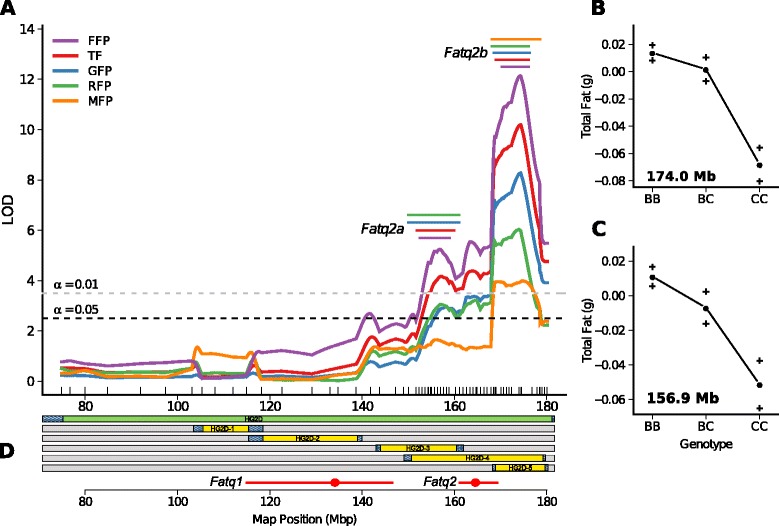
**QTL map for body fat traits on mouse chromosome 2. (A)** Interval mapping LOD score profile for Femoral (FFP), Gonadal (GFP), Mesenteric (MFP), and Retroperitoneal (RFP) Fat Pads and the sum of all fat pads referred to as Total Fat (TF). Horizontal dashed black and gray lines represent LOD threshold value for α = 0.05 and α = 0.01 after 1000 permutations, respectively. Since each threshold differed among fat pads, only the highest thresholds are plotted. The tick marks above the *x-axis* represent the genotyped SNP and microsatellite markers. **(B)** Genotypic effects on Total Fat at the peak location of *Fatq2b* at 174 Mb. **(C)** Genotypic effects on Total Fat at 156.9 Mb. The genotypes codes are BB = *b6/b6; BC = b6/cast; CC = cast/cast* **(D)** Subcongenic strains used for the fine mapping of *Fatq1* and *Fatq2*. Reported locations of *Fatq1* and *Fatq2* are displayed below the congenics (circle indicates location of the peak) [9].

Additive effects for each subcongenic strain were estimated by multiple linear regression as described in the supplemental methods (Additional file 1). Significant *additive* effects of CAST alleles on Total Fat (TF), Gonadal (GFP), Mesenteric (MFP), Retroperitoneal (RFP) and Femoral (FFP) fat pad weights were observed among the five subcongenic strains (Figure 1, Table 1). Differences in fat distribution were observed across the congenics (described in a separate paragraph), but in general CAST alleles tended to decrease all fat pad weights (Table 1). Thus only the Total Fat (TF: the sum of all fat pad weights) results are discussed in depth. The CAST alleles significantly reduced TF in HG2D-3, HG2D-4 and HG2D-5 (Figure 1, Table 1). Only the *additive* effect was significant (p < 0.009) in these strains, i.e. the *dominace* effect was not significant. A *sex × additive* (*sex × a*) interaction was detected only in HG2D-2, where CAST alleles increase TF in male mice. These phenotypic results support the location of *Fatq1* and *Fatq2* [9], and suggest a QTL × *sex* interaction for *Fatq1*. In contrast, phenotypic results of the subcongenics HG2D-3 and HG2D-5 demonstrate that what was previously described as Fatq2 is actually two QTL: Fatq2a and Fatq2b (Figure 2).

Farber and Medrano [9] previously reported that these QTL are *sex-biased,* and in these subcongenics it is mainly observed in HG2D-3. The *additive* effect of CAST alleles was similar in males from HG2D and HG2D-4, whereas females of HG2D show a greater reduction in TF than any other strain. From a theoretical standpoint *additive* effects observed in HG2D should be the cumulative sum of the independent *additive* effects observed in the subcongenic strains (*a*_*HG2D*_ *= a*_*HG2D-1*_ *+ a*_*HG2D-2*_ *+ a*_*HG2D-3*_ *+ a*_*HG2D-4*_ *+ a*_*HG2D-5*_ *+ e*). HG2D females show a reduction of −0.175 g TF, 1.2x the sum of HG2D-3, HG2D-4 and HG2D-5 (−0.216g = −0.057g + −0.101g + −0.058g, respectively) suggesting an undetected transgressive QTL that may control the remaining −0.041g [10,11]. In contrast, HG2D males have a reduction of −0.099 g TF, which is roughly the equivalent to the sum of HG2D-2, HG2D-3, HG2D-4 and HG2D-5 (−0.0803g = +0.072g + −0.012g + −0.095g + −0.045g, respectively). The same can be said for HG2D-4, where a reduction of −0.095 g TF in HG2D-4 is roughly 1.3x the sum of HG2D-3 and HG2D-5 (−0.057 g = −0.012 g – 0.045 g), suggesting an additional QTL on HG2D-4 that accounts for the remaining −0.038 g (Additional file 2: Figure S1).

Differences in the distribution of body fat were also observed across strains. The HG2D-3 strain had significant reductions in GFP and MFP, whereas no significant differences were found in RFP and FFP between *cast/cast* and *b6/b6* littermates. In contrast, HG2D-4 and HG2D-5 show significant reductions in GFP, RFP and FFP, but no significant differences in MFP between *cast/cast* and *b6/b6* littermates. Thus, loci within the unique donor region of the HG2D-3 strain primarily regulate MFP since this is the only strain with significant differences in MFP. All other fat pads (GFP, RFP and FFP) are primarily regulated by the unique donor region of the HG2D-4 and its overlap with the HG2D-5 strain, suggesting that loci regulating these fat pads are contained within the overlapping regions of these congenics (Figures 1 and 2, and Table 1).

### Linkage analysis of *Fatq2*

Individual interval mapping of the four fat pads and Total Fat reveal the presence of two QTL. The strongest QTL affecting all fat pads was located in the overlap region of strains HG2D-4 and HG2D-5 at 174 Mb (LOD = 14, referred to as *Fatq2b*) with a 95% confidence interval (CI) between 170.3 and 175.7 Mb (Figure 2) and a second QTL affecting total body fat and body weight was identified with a peak at 155 Mb (LOD = 5.5, referred to as *Fatq2a*) within the overlap of the HG2D-3 and HG2D-4 strains (CI: 150 to 161 Mb) (Figure 2). These results suggest the presence of two QTL segregating in the HG2D-4 strain that explain the effects of *Fatq2*.

This locus was previously observed as one large QTL by Farber and Medrano [9], however, the fine mapping provides evidence that *Fatq2* is the result of two QTL that flank the peak of the original *Fatq2*. Thus, suggesting that the peak of *Fatq2* in the previous analysis was the combined effects of two independent QTL with *additive* effects on body fat (Figure 2). These two QTL reduce total fat in the non-overlapping HG2D-3 and HG2D-5 subcongenics (p < 0.01 for the additive effect of CAST alleles; p ≥ 0.05 when comparing both strains).

### Fine mapping *Fatq2b* to the “*Gnas imprinted locus*”

**Figure 3:**
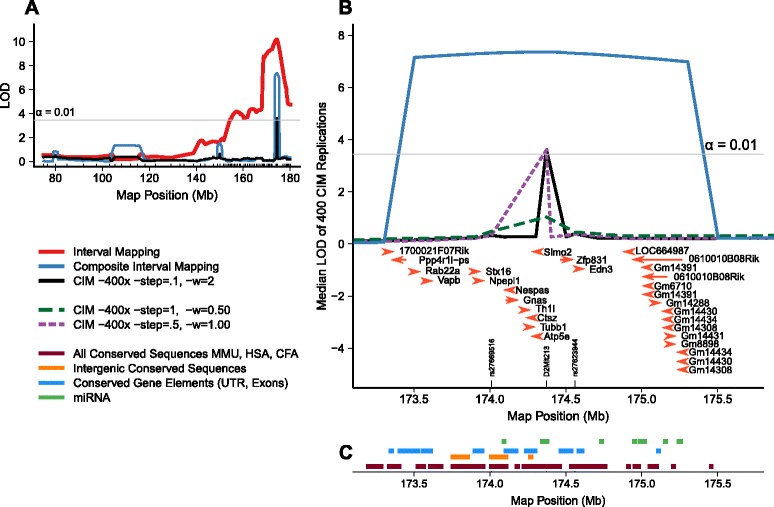
**High resolution QTL LOD profile for Total Fat of the 2.3 Mb CI of** ***Fatq2b*** **. (A)** Interval mapping and Composite Interval Mapping (CIM) LOD score profile for the HG2D region on Mouse Chromosome 2. **(B)** Magnification of *Fatq2b* (173.2 – 175.5 Mb). Median LOD score profiles after 400 replications of CIM. Two step and window sizes representative of all CIM parameter combinations are shown (Additional file 2: Figures S3 and S4) show median LOD scores for all parameter combinations). Genes are shown under the peak, with the direction of the arrows indicating sense of transcription. The horizontal gray line represents LOD threshold of IM value at α = 0.01 of 1000 permutations. **(C)** Dark red represents all the conserved non-coding sequences (CNS) in the *Fatq2b* region, orange lines represent intergenic conserved sequences that were analyzed for plausible promoters, transcription factors and gene elements. Light blue lines represent conserved gene elements among mice, humans and dogs. Green lines show the approximate locations of putative miRNA sequences that were identified by BLAST (Positions are an approximation since an 81 bp fragment would be too small to visualize on the scale).

Fine mapping of Total Fat with Composite Interval Mapping (CIM) positioned the peak *Fatq2b* QTL at 174.3 Mb (*D2Mit213*), with a confidence interval ranging from 173.5 to 175.5 Mb encompassing the *Gnas* imprinted *locus*. The summary of the replications of Composite Interval Mapping (CIM) consistently suggest a 0.6 Mb interval between 174.01 (*rs27669516*) and 174.56 Mb (*rs27623944*) (Figure 3) as the peak of *Fatq2*. This consistency of the peak location of *Fatq2b* was maintained even after changing the window and step size to 1, 0.5 and 0.25; and 0, 1, and 0.5, respectively in a 3 × 3 factorial design (Figure 3; Additional file 2: Figures S3 and S4), as described in the *Fine Mapping* methods. These analyses define the peak of *Fatq2b* to a region containing 30 putative candidate genes that regulate body fat deposition (Table 2), of which 3 are associated with body weight, body fat, and energy metabolism [12-15].

**Table 2:** **Positional candidate genes for the** ***Fatq2a*** **and** ***Fatq2b*** **QTL**

***mm9***		**Symbol**	**HG2D microarray p-value** ^**1**^ & **(tissue)** ^**2**^	**SNP** ^**3**^	**Sift** ^**4**^		**KO Models** ^**5**^	
**Chr**	**Location (Mb)**				**Prediction**	**Score**	**KO**	**P**
2	170.56	*Dok5*	--	--	--	--	--	--
2	172.07	*Mc3r*	--	--	--	--	3	Bh, G/S, Ob, S
2	172.2	*Cstf1*	--	*rs27646875*	deleterious	0	--	--
2	172.69	*Bmp7*	--	--	--	--	3	S, G/S, Eg, M/A
2	172.98	*Pck1*	--	--	--	--	4	Ob, M/A, G/S
2	173.03	*Zbp1*	--	--	--	--	1	--
2	173.34	*1700021F07Rik*	0.0220^(A)^	--	--	--	--	--
2	173.44	*Ppp4r1l-ps*	0.0035^(B)^	--	--	--	--	--
2	173.51	*Rab22a*	≤0.0001^(ABL)^	--	--	--	--	--
2	173.59	*Vapb*	≤0.017^(BL)^	--	--	--	--	--
2	173.91	*Stx16*	--	--	--	--	--	--
2	173.94	*Npepl1*	≤0.0050^(BL)^	--	--	--	--	--
2	174.11	Nespas**	--	--	--	--	1	PL, G/S, Bh
2	174.14	*Gnas*	≤0.0037^(AB)^	*rs28297630*	deleterious	0.03	12	EL, PL, G/S, Mt, Ob, DA
				*rs28297631*	deleterious	0.01		
				*rs28297633*	deleterious	0		
				*rs6313545*	deleterious	0.02		
				*rs6314659*	deleterious	0		
2	174.25	*Th1l*	--	--	--	--	--	--
2	174.26	*Ctsz*	0.0020^(B)^	*rs16789971*	deleterious	0.02	3	Tm
2	174.28	*Tubb1*	--		--	--	--	--
2	174.29	*Atp5e*	≤ 0.0021^(ABL)^	*rs28261286*	deleterious	0.01	--	--
2	174.29	*Slmo2*	0.0800^(B)^	--	--	--	--	--
2	177.75	*Etohi1*	--	*--*	*--*	*--*	--	--

**Antisense gene selected from MGI.

^1^P-values correspond to a comparison between HG2D Cast/Cast vs HG2D B6/B6 littermates (GSE22042) . The highest p-value is shown if multiple tissues were selected.

^2^Tissue: A = Gonadal White Adipose; B: Whole Brain; L: Liver. Genes without p-value had p > 0.1.

^3^SNP from dbSNP 128 contained in each gene with a deleterious Sift Prediction.

^4^SIFT prediction output from the ENSEMBL 76 Variation data set. Input variants were the 17,310 and 14,920 SNP variants contained between the CI interval of Fatq2a (170.5 to 173.03) and Fatq2b (173.3 and 175.6), BioMart settings were set to filter variants containing a tolerated and deleterious prediction between CAST/EiJ and C57BL/6J.

^5^KO = Number of Knockout Models; P = Phenotype; Bh = Behaviour; G/S = Growth and Size; Ob = Obesity related phenotype; S = Skeletal phenotype, Eg = Embriogenesis; M/A = Mortality/Aging; PL = Postnatal Lethality; EL = Embryonic Lethality; Mt = Metabolism; DA = Developmental Abnormalities; Tm – Tumorigenesis. Phenotype information and number of KO was obtained from MGI (revised March 2010).

### Differential expression of *Fatq2b* positional candidates

**Figure 4:**
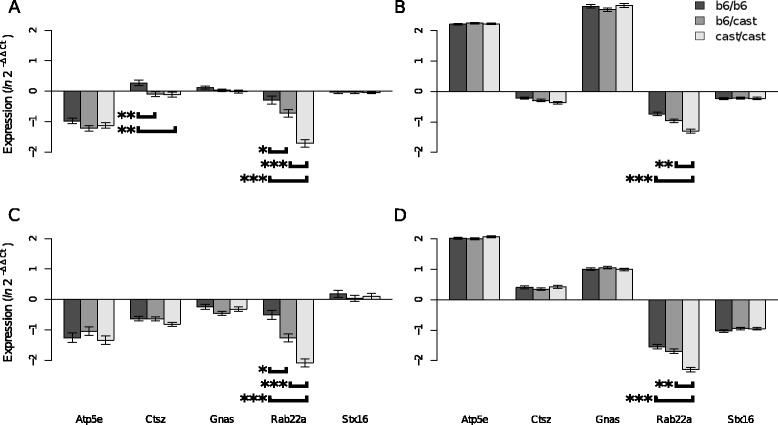
**Differential expression of** ***Fatq2b*** **candidate genes in whole brain and gonadal fat pad.** The diagram shows the relative gene expression profiles for *Atp5e*, *Ctsz*, *Gnas*, *Rab22a*, *and Stx16* for HG2D-4 (left) and HG2D-5 (right) in whole brain **(A, B)** and Gonadal Fat Pad (GFP) **(C, D)**. Differential expression of *Ctsz* in whole brain of was stronguest in HG2D-4 males (data not shown); though both sexes were differentially expressed (p < 0.05). *Rab22a* was differentially expressed in both whole brain and GFP in both the HG2D-4 and the HG2D-5 strains (p < 0 .001). Brackets represent the comparisons being made with the corresponding p-value *p = 0.05, **p < 0.01, and ***p < 0.001.

The QTL *Fatq2b* contains 14 known genes, and 16 additional transcripts all of which can be considered as putative candidate genes. Verdugo et al. identified *Atp5e, Ctsz, Gnas* and *Rab22a* as differentially expressed in adipose tissue and/or brain between B6/B6 and HG2D homozygous congenics (Table 2) [16]. We measured gene expression in brain and GFP using real time qPCR (RT-qPCR) with SYBR green in the four genes identified by Verdugo et al. (*Atp5e, Ctsz, Gnas* and *Rab22a*) and also in *Stx16*. Results show that *Ctsz* was differential expressed between *b6/b6* and *cast/cast* genotypes in brain tissue from the HG2D-4 strain only (p < 0.05) (Figure 4). *Rab22a* showed differential expression among genotypes in both brain and GFP tissues in both the HG2D-4 and HG2D-5 strains (p < 0.001) (Figure 4). All other tested genes did not show differential expression among genotypes in either tissue.

### Identification of putative micro RNA and transcription factor binding sites in *Fatq2b*

A screen for micro RNA (miRNA) in the entire 2.3 Mb genomic sequence yielded 14 miRNA sequences between 174.09 and 175.25 Mb (Figure 3C; and Table 3). However, no putuative miRNAs were located within intergenic conserved non-coding sequences using parameters described in the Methods.

**Table 3:** **BLAST results for miRNA screening of the 2.3 Mb confidence interval of** ***Fatq2***

***Chr***	***miRNA accession***	***Start (Mb)***	***End (Mb)***	***Identity (%)***	***Alignment length (bp)***	***Mismatches***	***Gap openings***	***E-value***
2	MI0000398	174.09301	174.09309	100	82	0	0	2.00E-37
2	MI0000969	174.09301	174.09309	96.34	82	3	0	3.00E-30
2	MI0014701	174.34603	174.34613	95.05	101	5	0	8.00E-37
2	MI0014701	174.3774	174.3775	94.06	101	6	0	2.00E-34
2	MI0014701	174.73593	174.73603	94.06	101	6	0	2.00E-34
2	MI0014701	174.95281	174.95289	91.57	83	6	1	7.00E-19
2	MI0006299	174.98866	174.98878	97.52	121	3	0	2.00E-53
2	MI0014701	175.01783	175.01791	91.86	86	6	1	1.00E-20
2	MI0014701	175.14765	175.14774	91.86	86	6	1	1.00E-20
2	MI0014701	175.15558	175.15568	94.06	101	6	0	2.00E-34
2	MI0014701	175.15708	175.15718	94.06	101	6	0	2.00E-34
2	MI0014701	175.24521	175.24529	91.86	86	6	1	1.00E-20
2	MI0014701	175.25314	175.25324	94.06	101	6	0	2.00E-34
2	MI0014701	175.25463	175.25473	94.06	101	6	0	2.00E-34

The Match™ search for transcription factor (TF) binding sites showed 23 TFBS in the promoter sequences of *Rab22a*, *Gnas* and *Ctsz*. The 3000 bp upstream of the *Gnas* transcription start site had one region at −2000 bp whose sequence is conserved among mice and human. This region contains three TF binding sites (HNF-1, C/EBP, and HLF) of which only HNF-1 has one SNP (Additional file 2: Figure S5). In humans, one SNP G(−1211)A on the functional promoter of *Gnas* showed an association with weight loss during a 7 day fasting period. Individuals with the GG genotype had a 5 ± 1.5 Kg weight loss, whereas the AA genotype had a 3.2 ± 1.2 Kg difference [17]. However, *Gnas* was not found differentially expressed between *cast/cast* and *b6/b6* homozygous in fat and whole brain from both HG2D-4 and HG2D-5 F2 littermates.

## Discussion

The present subcongenic experiments provides strong evidence that the *Fatq2* QTL, previously reported by Farber and Medrano [9], is composed of two QTL each with small effects. Congenic F2 intercrosses of five subcongenic strains with overlapping MMU2 donor regions were used to fine map *Fatq2b,* and in combination with high density genotyping in a large population allowed reducing the size of the QTL peaks to 2.3 Mb intervals and the identification of 22 positional candidate genes. Differential expression experiments reported here and known mouse knockout phenotypes suggest *Rab22a* and *Gnas* as candidate genes for the *Fatq2b* QTL, though, additional experiments are required to confirm any gene as the causal gene for *Fatq2b*.

The original peak of *Fatq1* mapped to 136 Mb [9], within the donor region of the HG2D-2 strain. Our results suggest that CAST alleles in this region increase total fat in congenic males by 0.072 ± 0.32 g (p = 0.03) (Table 1). Other fat QTL, such as *Aibl*, *Epfp1*, *Mob5*, *and Scfq1* have also been localized to this region [18-20]. However, the effects of the latter QTL on body fat were much larger than the effects observed in the HG2D-2 strain and were not sex dependent. The *Fatq1* QTL was originally identified by Farber and Medrano [9] in a population of 270 F2 mice from the HG2D congenic strain. This data was merged with the subcongenic data since the HG2D congenic is comparable to our five strains and a small peak at 140 Mb for FFP (LOD = 2.7), corresponding to *Fatq1* (Figure 2)*.*

The original peak of *Fatq2* in Farber and Medrano [9] maps to 164 Mb, corresponding to the unique donor region of the HG2D-4 congenic. However, the present analysis suggests that the *Fatq2* QTL is the product of two independent QTL; the first QTL located at 156.9 Mb, *Fatq2a*, and a the second QTL at 174 Mb, *Fatq2b.* (Figure 2). Results from the HG2D-4 congenic show decreases in GFP, RFP and FFP, consistent with the effects of *Fatq2.* Subcongenic analysis of this region suggests that the effects of CAST alleles on fat pad weights are greater in the HG2D-4 strain than in the HG2D-5 strain, consistent with the hypothesis that alleles present in the overlap region of the HG2D-4 and the HG2D-5 strain affect these traits. However, this does not completely explain the reductions in body fat by CAST alleles in HG2D-4 (Figure 1). The significant decrease of TF observed in the HG2D-3, HG2D-4 and HG2D-5 strains is likely the result of cumulative allelic effects on individual fat pads each controlled by more than one QTL (Figure 1).

The combined results from the HG2D-3 and HG2D-4 strain suggest that GFP and RFP are affected by loci present in overlap region of these strains (Figure 1), and MFP is affected by loci within the unique region of the HG2D-3 strain as HG2D-4 mice did not show significant differences in MFP. Furthermore, CAST alleles present in the HG2D-3 strain reduce GFP in female mice and CAST alleles from the HG2D-4 strain reduce GFP equally in both sexes (Table 1). This suggests a sex-specific regulation of affecting GFP among loci of the *Fatq2a* QTL present in the unique region of HG2D-3 and HG2D-5. The combined results strongly support the notion that *Fatq2* is not the result of a single QTL with one large effect, but rather the combined cumulative effect of at least two QTL each with a smaller contribution to TF, shown by the reduced TF of *cast/cast* mice from the HG2D-3 and HG2D-5 strains (Figure 2). With the current panel of subcongenic strains the presence or absence of a third QTL at 163.5 Mb –the location of the original *Fatq2* QTL cannot be confirmed as it is not uniquely isolated with a subcongenic strain.

The physical limit of *Fatq2b* is the HG2D-5 donor region, which spans MMU 2 from 168.5 to 178.5 Mb. Though, by integrating results from the linkage analysis, the most significant region is a 2.3 Mb interval between 173.3 Mb and 174.6 Mb. This region contains 22 known genes, of which gene expression of *Rab22a*, *Stx16*, *Atp5e*, *Gnas* and *Ctsz* were quantitated by RT-qPCR in GFP and whole brain from 60 HG2D-4 F2, and 60 HG2D-5 F2 mice. *Ctsz* showed differential expression in brain of HG2D-4 mice only, whereas *Rab22a* showed differential expression in both tissues and both strains analyzed (Figure 4). The region proximal to the peak of *Fatq2b*, between 168.7 to 173.3 Mb, contains 27 predicted genes, 23 known genes, 13 Riken transcripts and one microRNA. From the 23 known genes and 13 Riken transcripts; *Dok5*, *Mc3r, Bmp7,* and *Pck1* have been associated with obesity [21-24]. The region distal to the *Fatq2b* peak, between 174.6 and 178.5 Mb, has a segmental duplication that may be polymorphic in copy number between C57BL/6J and CAST/EiJ.

Differential expression of the genes listed above was first checked in microarrays between *cast/cast* congenic and control littermates. It is known that microarrays have limitations for QTL gene discovery, therefore, other positional candidate genes should not be discarded based on expression data alone [16]. In this study data from microarrays was used for an initial screening of differentially expressed genes that were later analyzed by RT-PCR in this study, but untested genes are still considered as plausible candidates.

Fine Mapping analyses, combined with the HG2D microarray and the RT-qPCR expression data in whole brain and GFP, suggest *Rab22a* as a plausible candidate for the *Fatq2b* QTL affecting body fat. However, published literature also shows body weight and obesity phenotypes between *Gnas* knockout and control mice [25], and has been implicated with performace traits in cattle [26], and carcass quality in pigs [27]. Other possible explanations for the observed phenotypic variation are possible SNP in the regulatory regions of the genes or variation at the transcription factor binding sites that may change the affinity of the transcription factor to regulate a gene [28]. With our current data it is not possible to discard genes outside the QTL peak that remain within the HG2D-5 congenic. However, the replicated CIM approach encourages further analysis of a 0.6 Mb region around *D2Mit213.* Though, the accuracy of replicating CIM to positionally clone individual genes has not been fully investigated, this method prioritized seven genes among the 51 known genes contained in the HG2D-5 strain. Several sources contributed information to the fine mapping of *Fatq2b* and support the use of this approach as an entry point to prioritize candidate genes in a QTL with a high density of genes.

The statistical methods IM and CIM used to fine map *Fatq2b* have several limitations when applied to mapping using subcongenic strains [29]. These are 1) the inability to detect epistasis, 2) the generation of ghost QTL, and 3) over fitting the analysis using too many markers as covariates. The inability to detect epistasis is caused by changing the native genotypes of interacting genes in the engineered panel of subcongenics, thus, changing the interaction itself or confounding the epistatic nature of the interaction to an additive effect in the individual subcongenics. Furthermore, any epistatic interactions with background alleles outside the congenic region are undetectable. The generation of ghost QTL is of major concern. However, this is unlikely in this experiment as the two identified QTL are observed in two independent subcongenics. Though, at this stage it is still unknown if a single gene is responsible for each QTL or if it is the cumulative effect of several small effects from many genes. Lastly, over fitting the QTL model by adding too many covariate markers. Over fitting the CIM may result in the increase of false positives. For this study, three covariate markers were used in all CIM analysis. Decreasing to two markers or increasing up to five markers did not have a significant effect in the results presented here.

The novel approach of replicated CIM and using a summary statistic of the LOD scores to generate a LOD profile has not been reported in the scientific literature. This approach averages the results of multiple CIM runs each with different imputation of missing markers and with unique covariate markers, resulting only one or very few markers with detectible signals after. Changing parameters such as step size and window size provides an assesment of the robustness of the location of the peak. However, once LOD scores are averaged, only those robust markers, consistently having high LOD scores, will result in a peak in the LOD profile. This can explain why only the strongest QTL, *Fatq2b*, was able to retain median LOD score of 3 after the 4,000 replications of CIM. Despite that this method has not been used elsewhere and the lack of alternative methods for subcongenic strains, the results obtained by this approach suggest further investigation of the “*Gnas* imprinted locus” and the effects that CAST alleles have on obesity at this locus.

The 0.6 Mb peak of *Fatq2b* suggested by the replicated CIM is the combined result of having a large mapping population (1,990 F2 mice), a high density SNP panel (1 SNP/600 Kb), and the replication of CIM with several parameter combinations. Though, *Fatq2b* has several genes involved in energy metabolism, protein transport and GTP cell signaling; analyses of gene expression in five genes suggests *Rab22a* as candidates, and the obesity phenotype of the *Gnas* knockout suggests these two genes as strong candidates for *Fatq2b*, though further analyses are required to confirm and ascertain the biological mechanisms of these genes in order to understand how *Fatq2b* exerts its effects on body fat.

## Conclusions

An integrative approach that included congenic and subcongenic QTL analysis, a large mapping population, and high density genotyping successfully partition one major QTL, *Fatq2*, into at least two QTL, and fine mapped the *Fatq2b* QTL on distal chromosome 2 to 174.5 Mb. The use of replicated composite interval mapping provided a 0.6 Mb region corresponding to the peak of *Fatq2b*. The confidence interval of *Fatq2b* contained several genes for which gene expression was measured, of these; *Rab22a* and *Gnas* are positional candidates that show differential gene expression for *Fatq2b*.

## Methods

### Mouse husbandry

Mice were housed in polycarbonate cages for a total of 6 weeks after weaning. Water and Purina LabDiet® 5008 chow (23.5% protein; Purina Mills Inc., St Louis MO) were offered *ad libitum.* A constant temperature (21°C ± 2°C) and humidity (40-70%) were maintained. Mice received 14 h of light, starting at 7:00 AM, and 10 h of dark. Mice were weighed at 14 days, and weaned at 21 days. Tail clips for DNA extractions were collected between 3 and 6 weeks of age. Animals were managed according to the guidelines of the American Association for Accreditation of Laboratory Animals and the Institutional Animal Care and Use Committee (IACUC).

### Development of congenic and subcongenic strains and F2 intercrosses

Five overlapping subcongenic strains were developed by selecting a five different recombinant mice from the HG2D F2 cross (Figure 1) [9]. Recombinant male mice were selected and used as founders of HG.CAST–(*D2Mit43–D2Mit103*) (referred to as HG2D-1), HG.CAST – (*D2Mit420 – D2Mit490*) (referred to as HG2D-2), and HG.CAST – (*D2Mit456 – D2Mit148*) (referred to as HG2D-5) strains. Recombinant female mice were selected and used as founders of the HG.CAST – (*rs27385876 – rs27703094*) (referred to as HG2D-4) and HG.CAST – (*D2Mit194 – rs27335891*) (referred to as HG2D-3) strains. Recombinant female mice were backcrossed to HG males to produce heterozygous congenic males. A male from these backcrosses was selected to propagate the strain.

To characterize the genotypic effects of the congenic region, F2 crosses were developed for each congenic strain. A total of 1,990 F2 mice were analyzed from five subcongenic strains, 384 for HG2D-1, 208 from HG2D-2, 382 from HG2D-3, 353 from HG2D-4 and 385 from HG2D-5; and 278 from (the founder HG2D founder strain previously analyzed by Farber and Medrano (2007b). Data from all congenics was merged and the analysis was carried out in the merged data set. Development and original analysis of the HG2D founder congenic strain is described in Farber and Medrano [9]. Sex ratios and genotype frequencies were tested with a χ^2^ test with 1 degree of freedom and did not differ from expected segregation ratios in any of the subcongenic strains (p > 0.05).

### Genotyping

Each subcongenic strain was initially genotyped with three microsatellite markers per strain (Additional file 3) using DNA isolated from Proteinase K digested tail clips and genotyped as described by Farber and Medrano [30]. The PCR reactions contained approximately 100ng of DNA (5 μl of diluted lysate), 0.1 units of *Taq* DNA Polymerase (ABI), 1X PCR Buffer (ABI), 1.5 to 2.0 mM of MgCl_2_ (Invitrogen, Carlsbad, CA), 0.17 mM of each dNTP (Invitrogen), 1μM of each primer in a total volume of 10 μl. PCR products were analyzed in 4% 0.5 TBE agarose gels containing 0.06 μg/ml EtBr.

To increase resolution for fine mapping after the initial QTL scans with microsatellite data, 366 mice with recombination events between 145 and 181 Mb from the HG2D founder, HG2D-3, HG2D-4 and HG2D-5 strains were genotyped with 48 SNP markers from 145 Mb to the end of MMU 2 at an average density of 1 SNP/~600 Kb based on the Genome reference mm9. Genotyping was carried out using the Sequenom MassArray® platform at GeneSeek Inc. (Lincoln, NE). DNA for SNP genotyping was purified by first incubating 100 μL of undiluted tail lysate with RNAse A at 37°C for 30 min. DNA was then precipitated with 200 μL of cold ethanol (EtOH), and centrifuged at 1300 RPM at 4°C for 15 min. The remaining pellets were washed with 300 μL of 70% EtOH, dried in a SpeedVac centrifuge for 4 min and resuspended in 32 μL of 10mM Tris pH 8. Purified DNA was diluted as necessary to keep the DNA concentration between 30 and 60 ng/μL.

### Phenotypic characterization

Phenotypes were selected based on their relevance to body weight (BW) and body fat. Mice were weighed to the nearest 0.1 g at 2, 3, 6 and 9 weeks of age and prior to sacrifice, 63 ± 4 days, (SAC). At sacrifice mice were anesthetized with isoflourane until they lost consciousness and were then placed over a grid without stretching to measure their lengths. Following sacrifice, dissection of the femoral (FFP; subcutaneous fat on the outer thigh), gonadal (GFP; interstitial fat surrounding the testis or uterus and ovaries), mesenteric (MFP; intraperitoneal fat surrounding the gastro-intestinal tract from the duodenum to the start of the rectum) and retroperitoneal (RFP; fat behind the kidney and along the lumbar muscle) fat pads. Tissue weights were collected immediately after dissection. Total Fat mass (TF) was calculated based on the data as the sum of all fat pads. Gonadal Fat Pad, whole brain, pituitary, gastrocnemius muscle and liver were snap frozen in liquid nitrogen and stored at -80^o^ C for future RNA extractions. Additionally collected trunk blood, and weighed liver, spleen, kidney, testis, empty carcass (skinned carcass without organs, fat, gastrocnemius, or tail), and gastrocnemius muscle; and femurs were measured to the nearest 0.1 mm with a Vernier scale. Mice were dissected in accordance with the University of California, Davis IACUC approved protocols. Genotype and phenotype data supporting this work are available at https://github.com/RodrigoGM/Mmu2QTL.git (doi:10.5281/zenodo.12793).

### RNA isolation and cDNA preparation

Total RNA was extracted from whole brain and GFP using TRIzol® (Invitrogen, Carlsbad, CA) according to manufacturer’s protocol. Whole brain and GFP were homogenized in 2 ml TRIzol® using a Mini BeadBeater–8 for 5 to 7 sec. For Real Time PCR, complementary DNA (cDNA) was prepared by taking 5 μg of total RNA from brain or GFP and incubating it with DNAse I (Ambion, Austin, TX) to remove any DNA contamination. Then first strand cDNA was synthesized using Superscript III® (Invitrogen, Carlsbad, CA) with poly-T and random primers according to manufacturer’s protocol. A final RNAse H (Ambion) incubation was done to eliminate single stranded RNA.

### Traits and statistical analyses

#### Characterization of subcongenic strains for body weight and body fat traits

Prior to QTL analysis, all subcongenics were independently analyzed using multiple linear regression models for GFP, MFP, RFP, FFP and TF traits. A detailed description of model selection, statistical analysis performed for each subcongenic strains is given in Additional file 1 and LS Means for all fat traits for males and females are in Table 1.

### QTL Analysis of body fat using congenic and subcongenic strains

#### Linkage mapping using interval mapping

QTL analyses were performed on the combined body fat data from the five HG2D subcongenic F2 intercrosses (described above), and the HG2D founder F2 intercross [9]. Initially, linkage analyses were performed in a stepwise procedure first using microsatellite genotypic data (Additional file 2: Figure S2), and then combining genotypic data from 48 SNPs to increase our mapping resolution of the peaks detected with microsatellites (see Genotyping methods). Covariates to correct for known environmental effects were selected based on our Subcongenic Analysis (Additional file 1).

Body fat phenotypes were adjusted for strain, sex and SAC weight. Residuals were used for the QTL analysis using the R/qtl package of the R Language and Environment [31,32]. Genotype probabilities were calculated using the *calc.genoprob* function at 0.1 Mb intervals. This is known as a step size and is further utilized in the fine mapping stage (see next section). Initial linkage analysis was performed using Interval Mapping (IM) on the adjusted phenotypes with the *scanone* function using Haley-Knott regression [33,34] over the physical map (NCBI37/mm9 assembly), since a genetic map could not be calculated accurately from the combined genotypic data. Confidence intervals were estimated with the *bayesint* function at a probability of 0.95. No specific *sex × QTL* interaction was detected. Significance thresholds were calculated by 1,000 permutations of each trait to estimate a LOD score to declare significance at α = 0.05 and α = 0.01 [35].

### Fine mapping QTL using composite interval mapping

To reduce the critical region of the Total Fat (TF) QTL composite interval mapping (CIM) [36,37] was used by applying the *cim* function of R/qtl using Haley-Knott regression, with a 2 Mb window and 0.2 Mb step size. Confidence intervals for the CIM were estimated using the *bayesint* function at a probability of 0.95. The *cim* function in R/qtl imputes missing genotypes using adjacent marker information and uses different markers as covariates in every run. This lead to an unstable location of the QTL peak in multiple sequential CIM runs. To accommodate this instability, the CIM analysis was replicated 400 times with a 2 Mb window (where three markers are used as covariates), and a 0.2 Mb step size in order to identify the most likely QTL peak location on the high density marker map. The Median LOD of the 400 replicates was used to summarize the replicates as the LOD score distribution at the tested marker were skewed due to changes in the imputed genotypes of missing markers being tested or used as covariates (data not shown). In addition, replicated CIM analyses for TF were repeated with a step size of 0, 1, and 0.5 Mb, and a window size of 1, 0.5, and 0.25 Mb arranged in a 3 × 3 factorial design to identify the most frequent location of the *Fatq2b* QTL peak. In total CIM was replicated 4,000 times (400 times with the initial parameters, and 400 times for each of the 9 factorial groups). The first replicates of CIM with a window of 2 Mb and step of 0.2 Mb serve as a baseline to compare the 3 × 3 factorial. This approach will be referred here as replicated CIM. A significance threshold value for the replicated CIM was not considered. This is because at this stage replicated CIM was used to pin point the most likely location of the peak within an already mapped QTL that is isolated within a 10 Mb congenic strain with phenotypic differences in body fat [38].

### Analysis of gene expression using microarrays

The results of Verdugo et al. [16], corresponding to the GSE22042 dataset were used to screen for genes with differential expression in the 2.3 Mb *Fatq2b* interval. This dataset is a microarray experiment where global gene expression of three tissues (GFP, whole brain, and liver) from 4 *cast/cast* and 4 *b6/b6* F2 mice that were non-recombinant for the entire HG2D congenic donor region was compared. The details of the analysis performed on the microarray data is described in Verdugo et al. [16]. We considered all genes within the confidence intervals of the *Fatq2b* QTL as differentially expressed genes if p ≤ 0.05 for the genotype effect. The p-values were not corrected for multiple comparisons since we are focused on specific genomic locations and wanted to maximize the number of genes to verify with real time qPCR (RT-qPCR).

### Analysis of differential expression in *Fatq2b* candidate genes using RT-PCR

To determine which of our primary candidate genes for *Fatq2b* were differentially expressed, gene expression was quantified in *Atp5e*, *Ctsz*, *Gnas*, *Rab22a* and *Stx16*, and two housekeeping *Gus* and *SDHA*. Gene expression was quantified using real time PCR in 20 *b6/b6* (7 females, 13 males), 20 *b6/cast* (11 females, 9 males) and 20 *cast/cast* (11 females, 9 males) mice of the HG2D-4 strain, and 21 *b6/b6* (11 females, 10 males), 20 *b6/cast* (10 females, 10 males) and 19 *cast/cast* (9 females, 10 males) of the HG2D-5 using FAST SYBR Green® or “Standard” SYBR Green® detectors for brain and GFP, respectively. Default PCR cycling times were used for each detector. Reactions were optimized to 1X FAST (or standard) SYBR Green® Master Mix (Applied Biosystems, Foster City, CA), 200nM of each primer, and 50 ng of cDNA in a total volume of 12 μL for the HG2D-4 samples and 20 μL for the HG2D-5 samples.

### Primer design

The mRNA sequence for each gene was re-sequenced with overlapping primers in two HG2D-4 homozygous mice, one cast/cast and one b6/b6 homozygous mouse. Accession numbers for the transcripts that were re-sequenced are shown in Additional file 4.

Primers used for SYBR green gene expression assays were designed from our CAST and B6 mRNA sequences using Primer Express® v.2.0 (Applied Biosystems, Foster City, CA) to ensure that the primer was not designed over a SNP not previously reported in dbSNP. These genes were used based on three lines of information: 1) Differential gene expression between C57BL/6J and CAST/EiJ in microarray experiments, 2) localization within the confidence interval from the replicated CIM analysis, and 3) association with growth or obesity based on the results of transgenic and/or knockout experiments. Any gene meeting at least one of these criteria was considered as a putative candidate.

The comparative Ct method was used to asses differential expression across genotypes [39]. Briefly, ∆∆Ct calculations were performed using the ddCt package from Bioconductor in the R: Language and Environment [40,41] as follows: the median Ct value of two housekeeping genes were used for the estimation of the ∆Ct, thus ∆Ct was estimated as Ct_Target_ – Ct_median(Gus, SDHA)_ in both strains. The reference sample for the ∆∆Ct was the mean Ct value between a pool of B6 Females and B6 Males, this was used only in the HG2D-4 strain. For the HG2D-5 a control sample was used instead. Finally, relative gene expression was estimated as 2^–∆∆Ct^. The 2^–∆∆Ct^ had different variances among genotypes, thus data was analyzed using a natural log *(ln)* transformation. Finally, to address differential expression across genotypes, gene expression as *ln*(2^–∆∆Ct^) was fitted to a linear model that accounted for the fixed effects of *sex* and *genotype*, and using PROC GLM in SAS® v. 9.2 (SAS Institute, Cary, NC). P-values were adjusted for multiple comparisons using the *SIMULATE* adjustment of LSMEANS statement using a sample size (*nsamp*) of 100,000 in SAS [42]. Comparisons between strains were not performed, as focus was placed to compare genotypes within each line.

### Analyses in conserved non-coding and promoter sequence analyses of *Fatq2b*

The entire 2.3 Mb genomic interval of *Fatq2b* was compared to the human, and dog genomes on the Vista Genome Browser to identify Conserved Non-coding Sequences (CNS) and conserved promoter regions and gene elements (UTR, exons) [43]. Also, the entire 2.3 Mb region and its intergenic CNS were screened for micro RNA (miRNA) from miRBase (Rel. 15) [44] using BLAST with default search parameters. Thresholds for considering a putative miRNA site were an e-value cut off of 0.01, alignment length greater than 80 bp and identity greater than 90%.

Three thousand base pairs upstream of the first exon or UTR were considered as the promoter region for each gene. These promoter sequences and the CNS within it were screened for putative Transcription Factor Binding Sites (TFBS) using the MATCH™ tool, which uses positional weight matrices from TRANSFAC® [45]. SNP and In/Dels polymorphic between CAST/EiJ and C57BL/6J were obtained from the Sanger Institute Mouse Genomes Project [46].

## Additional files

Additional file 1:
**Description of subcongenic analysis, model selection and estimation of LS Means for each congenic.**
Additional file 2: Figure S1. Additive effects of CAST alleles on total fat. Gray bars represent females and white represent males. **Figure S2.** Initial LOD profile for *Fatq2a* and *Fatq2b* based on 22 microsatellite markers. **Figure S3.** QTL map for total fat on mouse chromosome 2. **Figure S4.** LOD profiles of replicated composite interval mapping (CIM) for the Fatq2b QTL interval. **Figure S5.** Sequence analysis of *Rab22a*, *Gnas* and *Ctsz* promoter regions. Additional file 3:
**Table of microsatellite and SNP markers used to genotype congenic strains.**
Additional file 4:
**Table of primers used for gene expression analysis of** ***Atp5e, Ctsz, Gnas, Rab22a, and Stx16.***
