# Supplementary material for: Overlapping mouse subcongenic strains successfully separate two linked body fat QTL on distal MMU 2
Source: BMC Genomics. 2015 Jan 23;16(1):16. doi: 10.1186/s12864-014-1191-8 (PMC4308015; doi:10.1186/s12864-014-1191-8)
Supplement: Additional file 1: — Description of subcongenic analysis, model selection and estimation of LS Means for each congenic. [file 12864_2014_1191_MOESM1_ESM.pdf]

SUPPLEMENTAL METHODS

Subcongenic Analysis

Analyses were performed on 2, 3, 6 and 9 week body weights, NA, NT, tail and femur lengths, and liver, kidney, spleen, muscle, brain, TF, GFP, RFP, FFP and MFP weights. Phenotypes were classified as body weight, body size, organs and body fat measurements. Only body fat will be described here as most emphasis was placed on this phenotype. Other measurements were kept for further analysis.

Each subcongenic was analyzed for all phenotypes to determine the effect of CAST alleles in the donor region. Assumptions of normality and homogeneity of variances were tested with a Shapiro-Wilk and Levene's tests, respectively; and were visually inspected with histograms and box plots for data errors. Major deviations from normality were not observed in the phenotypic data, thus raw data was used. Phenotypic data (y) from each subcongenic was first fitted to a general linear model that accounted simultaneously for *additive* (a) and *dominance* (d) genotype effects, *sex* effects, *sex by additive* ($sex \times a$) and *sex by dominance* ($sex \times d$) genotype interactions, and correction with *additive* covariates (*cov*), e.g. Sex, SAC, Litter Size, Maternal Mating Weight, (Model 1). Interactions and covariates that were not significant were excluded from the model. Simple effects were analyzed if $sex \times a$ or $sex \times d$ interactions were significant ($p \leq 0.05$). This model contained *sex* as a dummy variable (Female=1; Male=0), a and d were estimated by multiple linear regression using *PROC GLM/Solution* statement of SAS® v9.1.3 (SAS Institute Inc., Cary NC), where a is the regression coefficients for the *additive* genotype effects of non recombinant subcongenic mice, and d is the dominance deviation from the mid parent. To estimate a, genotypes were assigned quantitatively as –1 for homozygous *b6/b6*, 0 for heterozygous *b6/cast*, and 1 for homozygous *cast/cast* genotypes (defined as g_1 in the model) (HG mice are on a C57BL/6J background, genotypes for HG background are denoted as *b6*, and

CAST alleles as *cast*). Similarly, to estimate *d,* genotypes were assigned quantitatively as 0 and 1 for homozygous and heterozygous genotypes, respectively (defined as g_2 in the model). In this case, *a* indicates the average effect of an allele substitution and *d* the deviation from the mid-parent of the heterozygous genotype (Falconer and Mackay 1996).

$$y_{ijkl} = sex_i + a(g_{1j}) + d(g_{2j}) + sex_i \times a(g_{1j}) + sex_i \times d(g_{2j}) + covariates_k + e_{ijkl} \qquad (Model\ 1)$$

After eliminating non significant interactions from the full model or *Model 1* each phenotype was analyzed with one of three models; *Model 2, Model 3 and Model 4*. To declare a significant genotype effect, p-values were adjusted with a Bonferronni correction for 5 comparisons to maintain an experimental error rate of 0.05. Thus, significant genotype effects were called with $p \leq 0.01$.

$$y_{ijl} = sex_i + a(g_{1j}) + d(g_{2j}) + e_{ijl} \qquad (Model\ 2)$$

$$y_{ijkl} = sex_i + a(g_{1j}) + d(g_{2j}) + covariates_k + e_{ijkl} \qquad (Model\ 3)$$

$$y_{jkl} = a(g_{1j}) + d(g_{2j}) + covariates_k + e_{jkl}\ [sexes_i\ analyzed\ separately] \qquad (Model\ 4)$$

Total Fat was analyzed using SAC and *sex×sac* as covariates (Lang et al. 2005; Stylianou et al. 2006); however both models had similar *a* and *d* effects of CAST alleles without changes in significance (data not shown). GFP, RFP, FFP and MFP were also corrected for *sex×sac* to maintain similar analysis of the components of TF. 6wk BW, 9wk BW and were corrected for *2wk BW* or *2wk BW*sex*. Other covariates that account for non-genetic factors, such as Litter Size (LS) and Dam's Mating Weight (MTW), were used only to analyze 6wk and 9wk BW in the

HG2D-4 strain only. Liver, Spleen, and Heart were corrected for SAC. Statistical procedures for subcongenic analyses were performed using the GLM procedure in SAS® v.9.1.3 (SAS Institute Inc., Cary NC).

REFERENCES

Falconer DS, Mackay TF (1996) Introduction to Quantitative Genetics, Fourth ed. (Edinburg: Pearson, Prentice Hall)

Lang DH, Sharkey NA, Lionikas A, Mack HA, Larsson L, Vogler GP, Vandenbergh DJ, Blizard DA, Stout JT, Stitt JP, McClearn GE (2005) Adjusting data to body size: a comparison of methods as applied to quantitative trait loci analysis of musculoskeletal phenotypes. J Bone Miner Res 20, 748-757

Perneger TV (1998) What's wrong with Bonferroni adjustments. BMJ 316, 1236-1238

Sankoh AJ, Huque MF, Dubey SD (1997) Some comments on frequently used multiple endpoint adjustment methods in clinical trials. Statistics in Medicine 16, 2529-2542

Stylianou IM, Korstanje R, Li R, Sheehan S, Paigen B, Churchill GA (2006) Quantitative trait locus analysis for obesity reveals multiple networks of interacting loci. Mamm Genome 17, 22-36
