# Supplementary material for: Overlapping mouse subcongenic strains successfully separate two linked body fat QTL on distal MMU 2
Source: BMC Genomics. 2015 Jan 23;16(1):16. doi: 10.1186/s12864-014-1191-8 (PMC4308015; doi:10.1186/s12864-014-1191-8)
Supplement: Additional file 2: Figure S1. — Additive effects of CAST alleles on total fat. Gray bars represent females and white represent males. Figure S2. Initial LOD profile for Fatq2a and Fatq2b based on 22 microsatellite markers. Figure S3. QTL map for total fat on mouse chromosome 2. Figure S4. LOD profiles of replicated composite interval mapping (CIM) for the Fatq2b QTL interval. Figure S5. Sequence analysis of Rab22a, Gnas and Ctsz promoter regions. [file 12864_2014_1191_MOESM2_ESM.pdf]

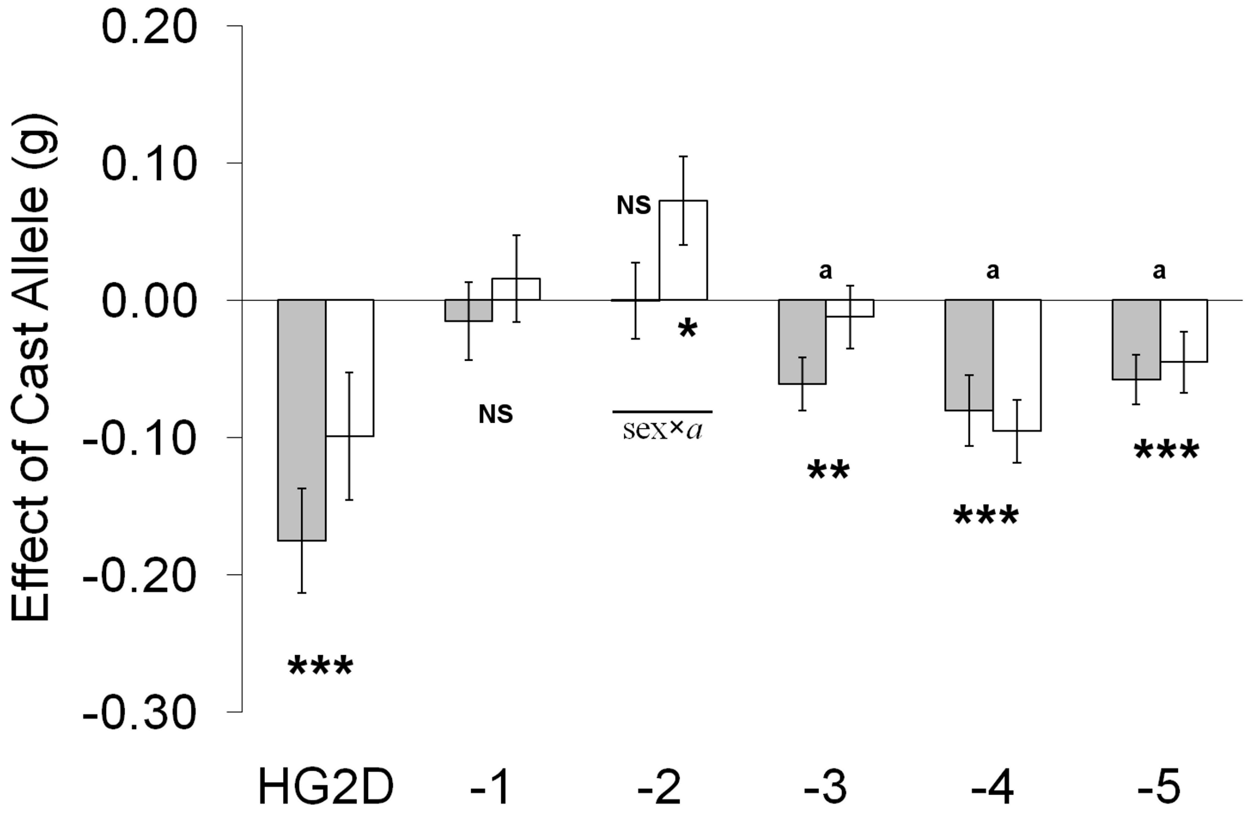

Figure S1. Additive effects of CAST alleles on total fat. Gray bars represent females and white represent males. Subcongenic strains are along the x-axis. A significant sex×a was observed in the HG2D-2 strain, where CAST allele only increase fat in males. Stars are representative for both males and females from the same strain, except in HG2D-2. Same letters above the strains 3, 4 and 5 indicates that the effect of CAST alleles was not significant among the strains. *: $p < 0.05$; **: $p < 0.01$; ***: $p < 0.001$; NS: Not Significant

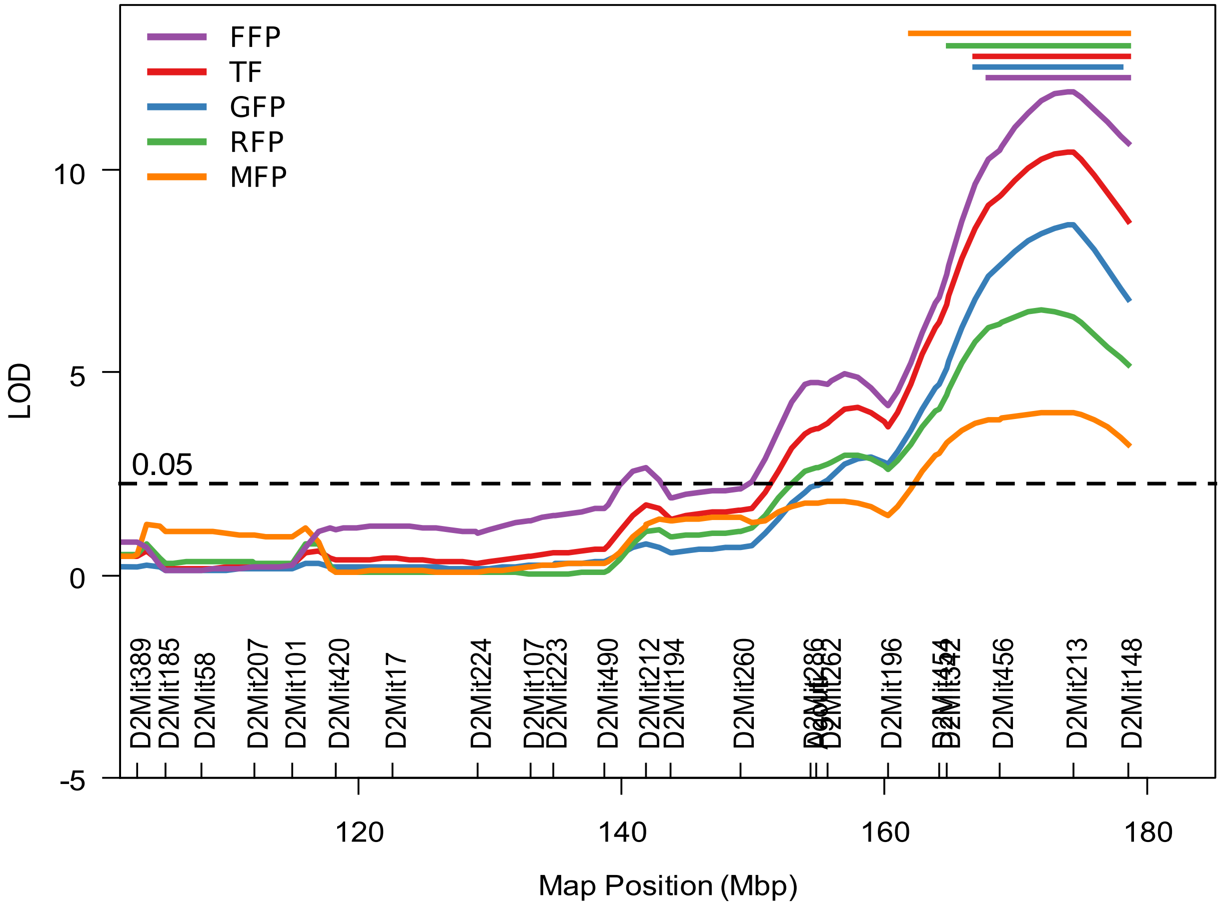

Figure S2. Initial LOD profile for *Fatq2a* and *Fatq2b* based on 22 microsatellite markers. It is observed that two peaks appear to be present, one small QTL over D2Mit262, and second larger peak on D2Mit213. These QTL are located on two subcongenic strains, HG2D-3 and HG2D-5, providing physical isolation of the QTL effects, and evidence that at least two QTL regulate total fat mass on distal MMU2.

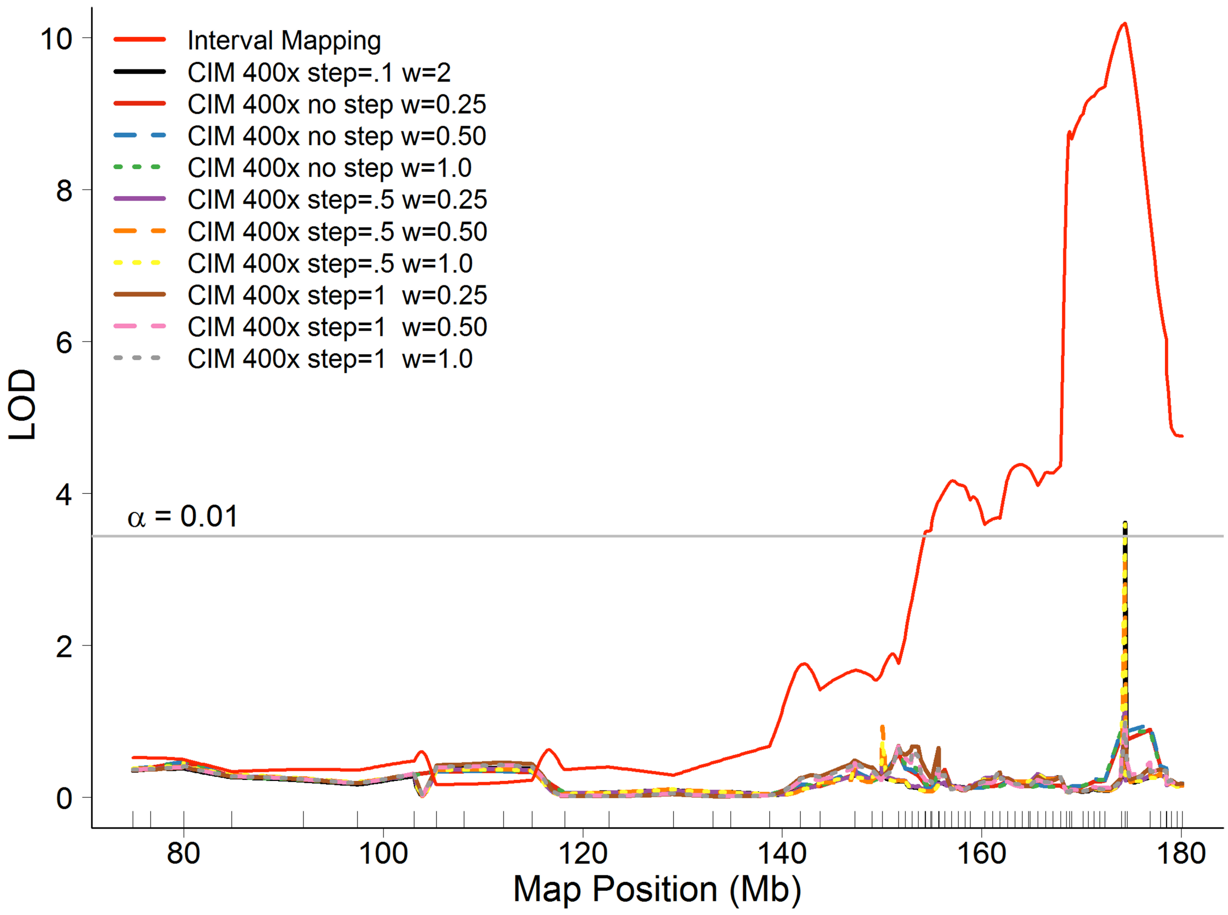

Figure S3. QTL map for total fat on mouse chromosome 2. Interval mapping suggests the presence of at least two QTL, one at 156.9 Mb and a second at 174.0 Mb, known as *Fatq2a* and *Fatq2b*. Both QTL were isolated in independent congenic strains (HG2D-3 and HG2D-5, respectively). Median LOD score for 400 replications of CIM suggest one strong QTL at 174.0 Mb. A statistical threshold for the replicated CIM was not considered. The presence of a QTL has been demonstrated by IM with a LOD > 10, and by CIM with a LOD > 7 coinciding with the peaks of the replicated CIM analyses. The replicated CIM was used to identify the most likely peak marker within the interval of the QTL and to prioritize candidate genes (Vieland 2006).

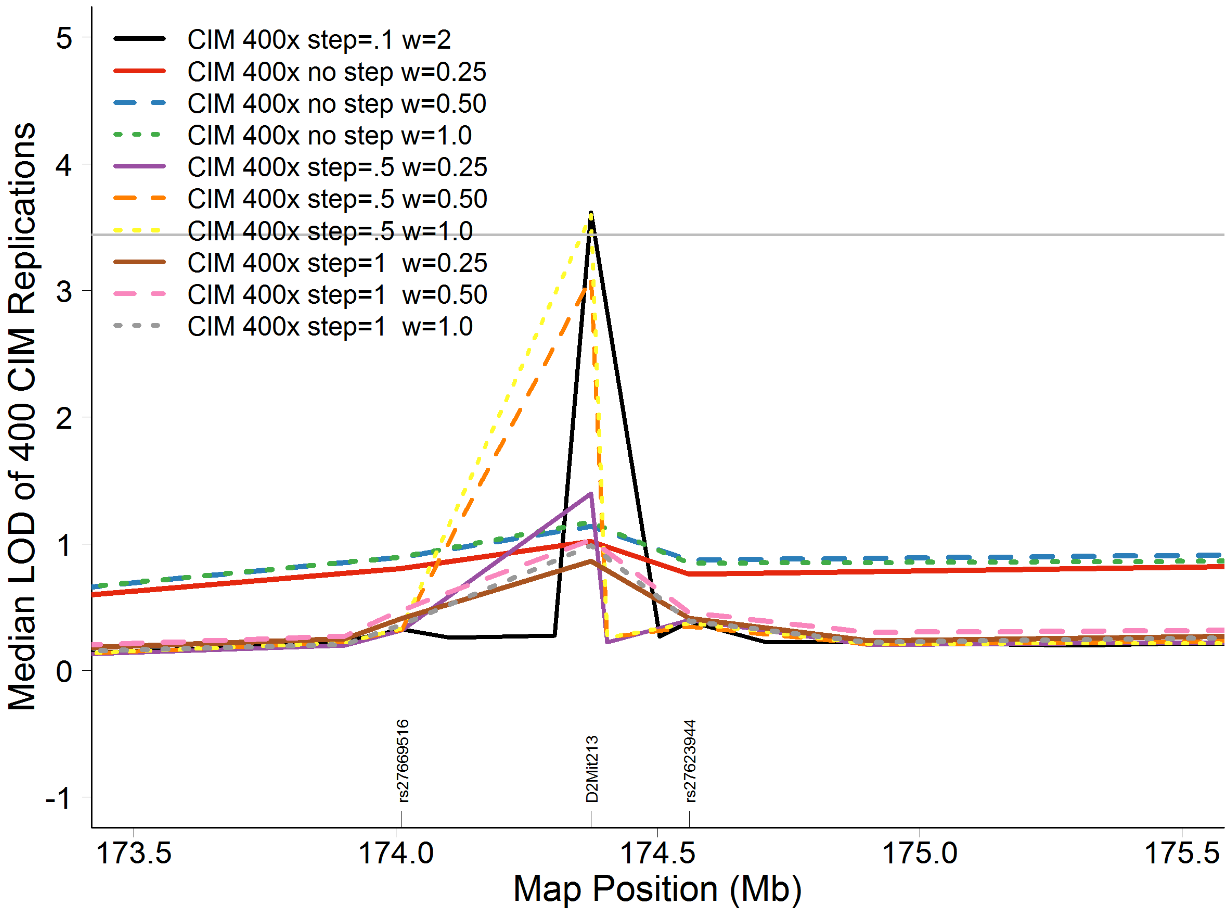

Figure S4. LOD profiles of replicated composite interval mapping (CIM) for the Fatq2b QTL interval. The diagram shows the median LOD score of 400 replications of CIM for 9 factorial combinations of window and step size. It is observed that all combinations suggest D2Mit213 as the peak marker, and suggest a critical region 0f 0.6 Mb for Fatq2b. For this study the 0.6 Mb region was considered as the peak of the QTL. This region contains 22 genes, of which *Rab22a*, *Gnas*, and *Ctsz* were considered as primary candidates. All other genes are not discarded as candidates but are of second priority for further experiments.

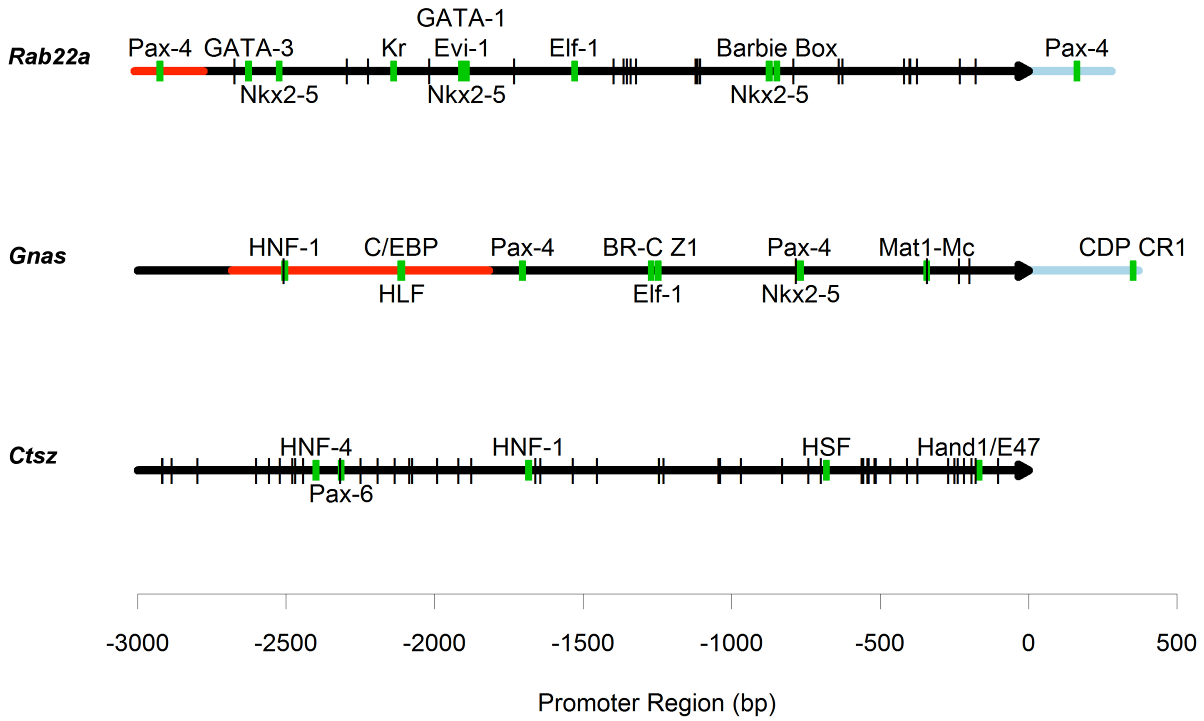

Figure S5. Sequence analysis of *Rab22a*, *Gnas* and *Ctsz* promoter regions. The black lines depict 3000 bp downstream of the genomic sequence of *Rab22a*, *Gnas* and *Ctsz* promoters; zero denotes the start position of each gene. The red region of the promoter indicates conserved non coding sequences (CNS) among mice, human, dog and Rhesus monkey. The green boxes represent motif for a transcription factor-binding site (TFBS) along the promoter. The name of the corresponding transcription factor is shown above or below the motif e.g. Pax-4, HNF-1, etc. Black tick marks on the promoter sequence represent the positions of polymorphic SNP and in/dels between C57BL/6J and CAST/EiJ. *Rab22a* and *Gnas* showed conserved regions in their promoters. Three TFBS, two on *Gnas* and one on *Ctsz* have a polymorphism within a 10 bp of the site. The *Rab22a* HNF-1 is in the conserved CNS of the promoter suggesting function and has one polymorphism which may explain the gene expression differences between CAST/EiJ and C57BL/6J.

References

Vieland, V. J. (2006). "Thermometers: something for statistical geneticists to think about." Hum Hered **61**(3): 144-156.
