# Supplementary material for: Overlapping mouse subcongenic strains successfully separate two linked body fat QTL on distal MMU 2
Source: BMC Genomics. 2015 Jan 23;16(1):16. doi: 10.1186/s12864-014-1191-8 (PMC4308015; doi:10.1186/s12864-014-1191-8)
Supplement: Additional file 3: — Table of microsatellite and SNP markers used to genotype congenic strains. [file 12864_2014_1191_MOESM3_ESM.pdf]

Additional Table 1. HG.CAST 2D subcongenic strains developed and their corresponding microsatellite and SNP markers, and PCR conditions.

Strain	***Microsatellite***	***[MgCl$_2$] mM***	***Location***		***Source***
			**Mb**	**cM**	
HG2D-1	*D2Mit185*	2.0	105.335860	47.5	MIT
HG2D-1	*D2Mit58*	2.0	108.099851	51.4	MIT
HG2D-1	*D2Mit101*	2.0	114.905325	52.5	MIT
HG2D-2	*D2Mit420*	2.0	118.203953	54.6	MIT
HG2D-2	*D2Mit107*	2.0	133.061498	61.2	MIT
HG2D-2	*D2Mit490*	2.0	138.721284	64.5	MIT
HG2D-3	*D2Mit194*	2.0	143.757317	81.4	MIT
HG2D-3	*rs27273819*		145.506914		Perlegen2
HG2D-3	*rs27325361*		147.258580		Perlegen2
HG2D-3	*D2Mit260*	1.5	149.008060	83	MIT
HG2D-3	*rs27372237*		150.007335		Perlegen2
HG2D-3	*rs27350314*		150.998686		Perlegen2
HG2D-3, and -4	*rs27385876*		151.669008		Perlegen2
HG2D-3, and -4	*rs27343750*		152.330044		Perlegen2
HG2D-3, and -4	*rs27352724*		152.991660		Perlegen2
HG2D-3, and -4	*rs27343642*		153.649790		Perlegen2
HG2D-3, and -4	*D2Mit286*	2.0	154.338414	87.0	MIT
HG2D-3, and -4	*rs27365903*		154.312195		Perlegen2
HG2D-3, and -4	*rs27326115*		154.978855		Perlegen2
HG2D-3, and -4	*D2Mit262*	2.0	155.693206	87.0	MIT
HG2D-3, and -4	*rs27323847*		155.643661		Perlegen2
HG2D-3, and -4	*rs27321848*		156.305388		Perlegen2
HG2D-3, and -4	*rs27307998*		156.976171		Perlegen2
HG2D-3, and -4	*rs27358422*		157.638649		Perlegen2
HG2D-3, and -4	*rs27319627*		158.299081		Perlegen2
HG2D-3, and -4	*rs27306308*		158.858393		Perlegen2
HG2D-3, and -4	*rs27335891*		159.621255		Perlegen2
HG2D-4	*D2Mit196*	2.0	160.286752	92.0	MIT
HG2D-4	*rs28279021*		161.056198		Perlegen2
HG2D-4	*rs27317780*		161.808146		Perlegen2
HG2D-4	*rs28280230*		162.568440		Perlegen2
HG2D-4	*rs16787600*		163.332178		Perlegen2
HG2D-4	*rs27300434*		163.923855		Perlegen2
HG2D-4	*rs28240341*		164.844700		Perlegen2
HG2D-4	*rs27330151*		165.615450		Perlegen2
HG2D-4	*rs27298803*		166.381247		Perlegen2
HG2D-4	*rs27290252*		167.106665		Perlegen2
HG2D-4	*rs27322351*		167.891803		Perlegen2
HG2D-4	*rs27288813*		168.459905		Perlegen2
HG2D-4, and -5	*D2Mit456*	1.5	168.761938	86.3	MIT
HG2D-4, and -5	*rs27340088*		169.001154		Perlegen2
HG2D-4, and -5	*rs27622165*		169.568797		Perlegen2
HG2D-4, and -5	*rs6233743*		170.120252		Perlegen2

Strain	***Microsatellite***	***[$MgCl_2$] mM***	***Location***		***Source***
			**Mb**	**cM**	
HG2D-4, and -5	*rs27618585*		170.675178		Perlegen2
HG2D-4, and -5	*rs28289712*		171.240389		Perlegen2
HG2D-4, and -5	*rs27289118*		171.786730		Perlegen2
HG2D-4, and -5	*rs27631793*		172.345636		Perlegen2
HG2D-4, and -5	*rs27646938*		172.888191		Perlegen2
HG2D-4, and -5	*rs27644321*		173.450373		Perlegen2
HG2D-4, and -5	*rs27669516*		174.011386		Perlegen2
HG2D-4, and -5	*D2Mit213*	1.5	174.372768	105.0	MIT
HG2D-4, and -5	*rs27623944*		174.559892		Perlegen2
HG2D-4, and -5	*rs27634370*		175.001750		Perlegen2
HG2D-4, and -5	*UCDSNP2*		176.595657		UC Davis
HG2D-4, and -5	*rs27650182*		176.860174		Perlegen2
HG2D-4, and -5	*rs27650079*		177.339866		Perlegen2
HG2D-4, and -5	*rs27652370*		177.890297		Perlegen2
HG2D-4, and -5	*rs27703094*		178.449714		Perlegen2
HG2D-5	*D2Mit148*	1.5	178.535307	105.0	MIT
Outside congenic	*rs27683474*		179.000149		Perlegen2
Outside congenic	*rs27648414*		179.546670		Broad1
Outside congenic	*rs27681748*		180.104819		Perlegen2
