# Supplementary material for: Overlapping mouse subcongenic strains successfully separate two linked body fat QTL on distal MMU 2
Source: BMC Genomics. 2015 Jan 23;16(1):16. doi: 10.1186/s12864-014-1191-8 (PMC4308015; doi:10.1186/s12864-014-1191-8)
Supplement: Additional file 4: — Table of primers used for gene expression analysis of Atp5e, Ctsz, Gnas, Rab22a, and Stx16. [file 12864_2014_1191_MOESM4_ESM.pdf]

Additional Table 2. Real Time PCR primers for differential expression with SYBR Green.

| ***Name*** | ***Orientation*** | ***Sequence*** | ***Fragment Size*** | ***Accession No.*** |
|---|---|---|---|---|
| *Atp5e* | *F* | *ACTTCGGGCAGCAGCATAA* | *95* | *NM_025983.3* |
| | *R* | *CCCACATGCTTCACCTTCAA* | | |
| *Ctsz* | *F* | *AGTACTGGATTGTCCGAAATTCATG* | *91* | *NM_022325.4* |
| | *R* | *ACCTGTGCCTCCCTTGTAGGT* | | |
| *Gnas* | *F* | *CAAGGAGCAACAGCGATGGT* | *92* | *NM_022000.2* |
| | *R* | *TCATGGCGGCCACAATG* | | |
| *Rab22a* | *F* | *CACTGGACGCCACCTCATG* | *127* | *NM_024436.2* |
| | *R* | *CCCTTTGCACGCCAGAGT* | | |
| *Stx16* | *F* | *CGTGTGAATACGTGCTTTAGTGAA* | *111* | *NM_172675.2* |
| | *R* | *TTTAACATGCTGCTCAGACATTAGTG* | | |
| *Gus*[*] | *F* | *GTATGGAGCAGACGCAATCC* | *134* | *NM_010368* |
| | *R* | *GCTCTCCGACCACGTATTCT* | | |
| *SDHA*[*] | *F* | *CGGCAAATCTCAACTTGTCA* | *131* | *XM_127445* |
| | *R* | *CCTTGTAGTCTTTGGCAGAGC* | | |

* (Wong 2004)
